# Environmental factors and stochasticity affect the fungal community structures in the water and sediments of Hulun Lake, China

**DOI:** 10.1002/ece3.9510

**Published:** 2022-11-18

**Authors:** Yongquan Shang, Xiaoyang Wu, Xibao Wang, Huashan Dou, Qinguo Wei, Shengchao Ma, Guolei Sun, Lidong Wang, Weilai Sha, Honghai Zhang

**Affiliations:** ^1^ College of Life Sciences Qufu Normal University Qufu China; ^2^ Hulunbuir Academy of Inland Lakes in Northern Cold & Arid Areas Hulunbuir China

**Keywords:** aquatic fungal, Hulun Lake, ITS2, sediment, stochastic, water

## Abstract

Aquatic fungi form both morphologically and ecologically diverse communities. However, lake ecosystems are frequently overlooked as fungal habitats, despite the potentially important role of fungi in matter cycling and energy flow. Hulun Lake is a typical example of a seasonal glacial lake; however, previous studies have only focused on bacteria in this ecosystem. Therefore, in the current study, internal transcribed spacer ribosomal RNA (ITS rRNA) gene high‐throughput sequencing was used to investigate the fungal communities in paired water and sediment samples from the Hulun Lake Basin in China. A significant difference was found between the fungal communities of the two sample types. Across all samples, we identified nine phyla, 30 classes, 78 orders, 177 families, and 307 genera. The dominant phyla in the lake were Ascomycota, Basidiomycota and Chytridiomycota. Our results show that both water and sediments have very high connectivity, are dominated by positive interactions, and have similar interaction patterns. The fungal community structures were found to be significantly affected by environmental factors (temperature, chemical oxygen demand, electrical conductivity, total phosphorus, and pH). In addition, the dispersal limitations of the fungi affected the structure of the fungal communities, and it was revealed that stochasticity is more important than deterministic mechanisms in influencing the structure and function of fungal communities. This study provides unique theoretical support for the study of seasonally frozen lake fungal communities and a scientific basis for the future management and protection of Hulun Lake.

## Graphical abstract

The fungal community structures were found to be significantly affected by environmental factors (temperature, chemical oxygen demand, electrical conductivity, total phosphorus, and pH). In addition, the dispersal limitations of the fungi affected the structure of the fungal colonies, and it was revealed that stochasticity is more important than deterministic mechanisms in influencing the structure and function of fungal communities.

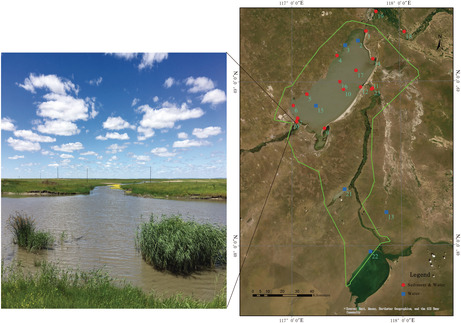

## 1 INTRODUCTION

Fungi are ubiquitous to all ecosystems and play an important role in maintaining ecosystem function and health (Cai et al., 2006; Grossart et al., 2019). They are major drivers of the global biochemical cycle and decomposers of animal and plant remains (Cerro‐Galvez et al., 2019; Maynard et al., 2019; Mhuantong et al., 2015). Fungi play a potentially important role in organic matter cycling and food web dynamics (Grossart et al., 2019). They can also be pathogenic and cause numerous diseases, which can seriously threaten global food security and human health (Head et al., 2014; Zhao et al., 2020). Despite the ecological significance of fungi, previous studies on microorganisms have mainly focused on the bacteria in lakes (Sadeghi et al., 2021; Shang et al., 2020; Shao et al., 2021), forests (Tian, He, et al., 2018; Zhu et al., 2020), oceans (Feng et al., 2009; Zhang, Liu, et al., 2021), and rivers (Qian‐Qian et al., 2021). Moreover, investigations on freshwater aquatic ecosystems, such as rivers and lakes, as main habitats of fungi have been limited (Khomich et al., 2017, 2018; Li et al., 2021). Consequently, there is an urgent need to extend our knowledge of community structures and the factors influencing the fungal community in these habitats.

In recent years, the significance of microbes has been revealed due to the continuous development of high‐throughput sequencing (Bahram et al., 2021). In addition, microbial diversity and several of its influencing factors have been discovered and understood (Zhang, Delgado‐Baquerizo, et al., 2021; Zhao et al., 2022). Fungal community composition can be affected by various environmental factors, such as salinity, total potassium, and altitude (Sheng et al., 2019; Zhao et al., 2019). Studies have also shown that the microbial community structures in natural waters reservoir (Yangtze River) are closely related to changes in environmental factors (Jiang et al., 2021). Tian, Zhu, et al. (2018) reported that altitude, mean annual temperature, carbon/nitrogen ratio, dissolved organic carbon, and total nitrogen are the best predictors of fungal community structure in freshwater lakes (Tian, Zhu, et al., 2018). Livermore and Mattes (2013) found that salinity is the main factor affecting estuarine fungal community (Livermore & Mattes, 2013). However, research on the factors influencing seasonal frozen‐lake fungal communities remains relatively scarce.

The assembly, structure, and function of fungal communities are important for water ecology (Nemergut et al., 2013). Studies have shown that microbial community changes are mainly influenced by two ecological processes: deterministic (niche‐based) and random (neutral) processes (Chase, 2010; Chase et al., 2011). Community building mechanisms have been investigated in various environments, including soils, oceans, lakes, and microplastic surfaces (Liu, Zhu, et al., 2020; Ortiz‐Alvarez et al., 2020; Sun et al., 2021; Tripathi et al., 2018). Studies have shown that the contributions of deterministic and random processes to microbial assembly may differ in terrestrial and aquatic ecosystems (Yang et al., 2016). There is ecological differentiation in lake water and sediments, but the relative contributions of random and deterministic processes to community aggregation in the two habitats remain unclear.

Hulun Lake is the fifth largest lake in China and the largest lake in the northern region of the country (Fang et al., 2018). It is rich in biodiversity and plays a unique role in regulating the regional climate, conserving water, protecting biodiversity, and maintaining the security of Northern China (Zhang, Chen, et al., 2021). The important role of microorganisms in lake ecosystems has been extensively studied (Zhang et al., 2020), but the focus has mainly been on bacterial communities. Consequently, research on fungi in lakes is limited, and almost no research has been conducted on the fungal communities in Hulun Lake (Li et al., 2021). Investigations on the fungal community structure and its influencing factors in Hulun Lake can enrich fungal resources and help maintain the healthy development of this ecosystem. As Hulun Lake is also a representative seasonal glacial lake, research conducted here will provide valuable reference data for similar ecosystems.

In this study, we used high‐throughput sequencing technology to investigate the diversity and community composition of fungi in the Hulun Lake Basin, focusing on the influence of abiotic factors on the fungal community and the construction mechanisms of the fungal community in the Hulun Lake Basin. The findings of the study could increase our understanding of the fungal communities in a seasonal frozen lake and how these communities respond to various abiotic factors; the knowledge will be necessary for the conservation and restoration of Hulun Lake biodiversity.

## 2 METHODS

### 2.1 Study area and sampling

The Hulun Lake region is located in the middle and high latitudes of the western Hulun Buir Plateau, in a semi‐arid grassland with a continental climate in the middle temperate zone. It has an average annual temperature of 0.3°C and is covered with ice for approximately 180 days annually (Hulun Lake Chronicle). This study was conducted in Hulun Lake (48°30′40″–49°20′40″N, 117°00′10″–117°41′40″E), which is in the Hulun Buir Steppe of the Inner Mongolian Autonomous Region of China (Inner Mongolia Hulun Lake to national nature reserve annals). It is 93 km long, with an average width of 32 km and circumference of 447 km (Hulun Lake Chronicle).

**FIGURE 1:**
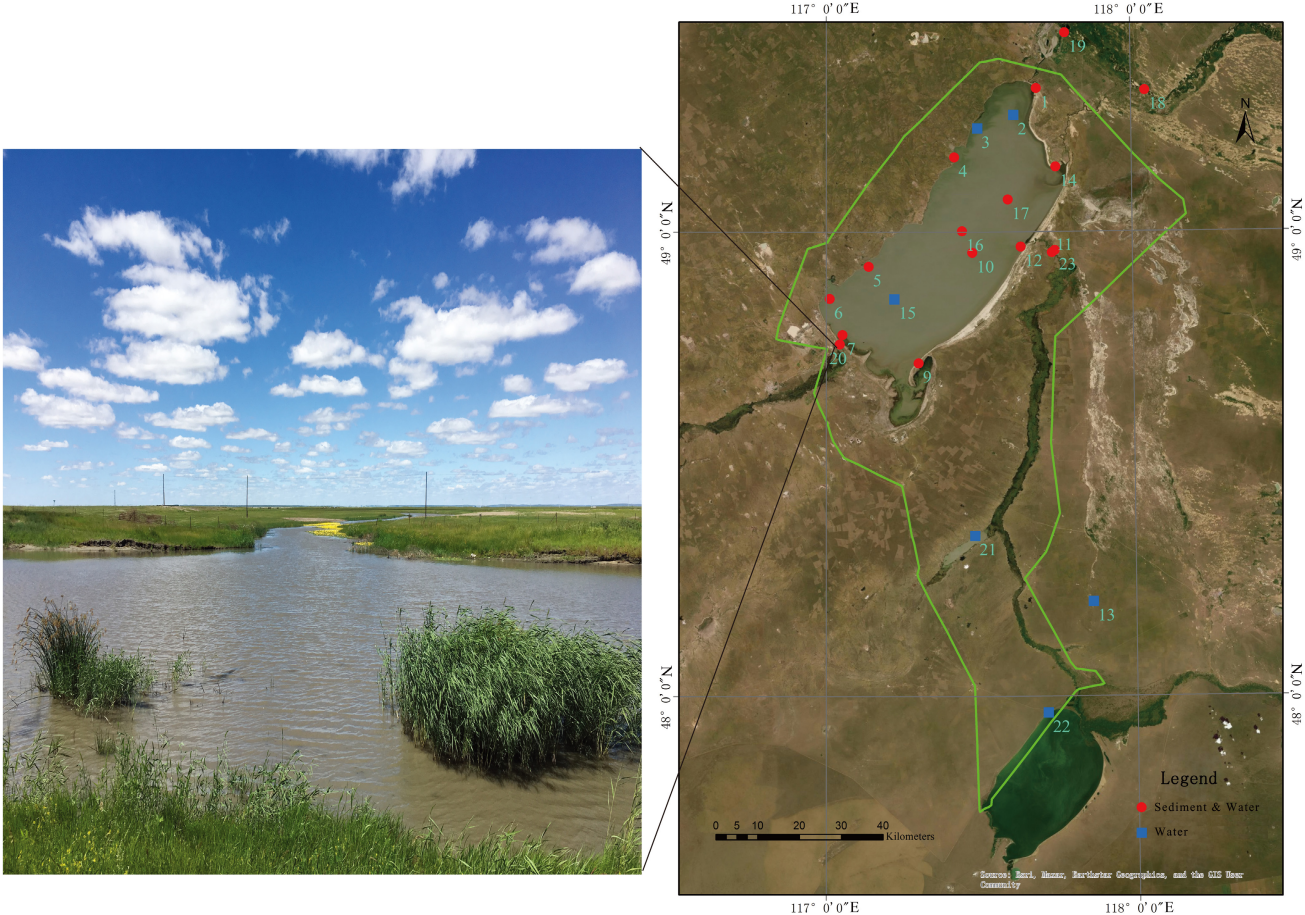
Distribution map of the summer sampling points in the Hulun Lake Reserve. The latitude and longitude of the sampling points are shown in Table S1. The light green line represents the Hulun Lake reserve.

A total of 38 samples (16 sediment and 22 water samples) were collected from 23 sites covering the entire Hulun Lake Basin in the summer of 2018 (Figure 1). Triplicate water samples (approximately 0.5 m below the water surface, and the upper and lower layers were sampled from areas with a water depth of >5 m) were collected from each site using a 2.5 L plexiglass water collector, mixed, then immediately stored in a 10 L sterile polyethylene bucket, and quickly transported to the laboratory. Based on the turbidity of the water, the sample was filtered through a 0.45 μm filter membrane (300–1000 ml), and the membrane was then preserved at −80°C. Three sediment replicates from each sample point were also collected, mixed, and stored at −80°C. To characterize the environment in each plot, we measured temperature, pH, total P, ammonia nitrogen (NH_4_^+^‐N), total N, electrical conductivity (EC), chemical oxygen demand (COD), and dissolved oxygen (DO), according to previously described methods (Shang et al., 2022).

### 2.2 DNA extraction and high‐throughput sequencing

Fungal DNA was extracted using a HiPure Soil DNA Kit (Magen) according to manufacturers' protocol. The ITS2 rRNA region was amplified using the PCR primers ITS3_KYO2 (GATGAAGAACGYAGYRAA) and ITS4 (TCCTCCGCTTATTGATATGC) (Wu et al., 2022). PCR amplification was conducted using previously described methods (Wu et al., 2022). Purified amplicons were standardized so that they were equimolar, then pooled and paired‐end sequenced (PE250) on an Illumina platform (Illumina Hiseq 2500) according to standard protocols. The method used is described in full in the Appendix S1.

### 2.3 Statistical analyses

The representative fungal OTUs are listed in Data S1. Based on the operational taxonomic unit (OTU) information, alpha diversity indices (observed number of OTUs [Sobs], Chao1, Simpson, Shannon, and Good's coverage) of the Illumina HiSeq sequencing data were analyzed using QIIME (version 1.2.11). The alpha index comparison between groups was calculated using Wilcoxon‐test in R (version 3.6.1, The R Project for Statistical Computing). The relative impact of the environmental parameters on alpha diversity was assessed using the Gbmplus software package (Guo et al., 2017). Principal co‐ordinate analysis (PCoA) of β‐diversity based on Bray–Curtis distances was conducted using the “vegan” package in R (version 3.6.1) to analyze fungal community similarity (Hartman et al., 2020). Permutation multivariate analysis of variance (PERMANOVA) and analysis of similarities (ANOSIM) of the Bray–Curtis and Jaccard distances were conducted to test for differences in the sediment and water samples. The relationship between the fungal communities and environmental factors was investigated using redundancy analysis (RDA) and correlation analysis.

Phylogenetic molecular networks (pMENs) were established using R for the water and sediment samples based on the random matrix theory (RMT) (Zhou et al., 2010). OTUs with the top 100 abundances were used to obtain the Spearman's rank correlation matrix. An identical similarity threshold (0.5) was used to construct networks for water and sediment samples. The networks were visualized using Gephi 0.9.1 (Bastian et al., 2009).

To explore the ecological process of community assembly, we used the β‐nearest taxon index (βNTI) and Bray‐Curtis‐based Raup‐Crick (RC_bray_) index to quantify the community assembly process (Stegen et al., 2013). We applied R to calculate the weighted beta nearest taxon index (Webb et al., 2002). A βNTI < −2 and βNTI > 2 indicated homogeneous and heterogeneous selections, respectively (Dini‐Andreote et al., 2015). If |βNTI| < 2, but RC_bray_ < −0.95, or RC_bray_ > 0.95, this suggested that community turnover was governed by homogenizing dispersal or dispersal limitation, respectively. In contrast, if |βNTI| < 2 and |RC_bray_| < 0.95, this suggested that community turnover is governed by undominated processes (Stegen et al., 2015).

Spearman correlation was employed to explore the correlation between environmental factors using SPSS software and significant correlations were visualized with the “ggcorrplot” package. One‐way analysis of variance was used to analyze the environmental parameters and abundance of the microbial communities. The correlation between the two matrices was determined by performing the Mantel‐test analysis (Pearson correlation, 999 permutations) using Vegan package in R (Hu et al., 2021).

## 3 RESULTS

### 3.1 Environmental parameters

The environmental parameters of Hulun Lake are listed in Table S1. The average sample temperature was 24.78 ± 1.65°C. Based on the pH, Hulun Lake was determined to be weakly alkaline (pH: 8.3–9.1). The P content of samples 5 and 16 exceeded that of all other samples tested in this study and were six times higher than that of the samples with the lowest P content. For the N and P content values, there was a large difference in their spatial distribution between the various sampling sites. In addition, the temperature was found to significantly negatively affect P (*p* < .05), NH_4_^+^‐N, EC, and COD, while the pH significantly positively affected the COD and EC (*p* < .05; Figure S1).

### 3.2 Fungal community composition and diversity in water and sediments

A total of 5,030,493 tags of raw data were obtained from the sequencing machine. After removing low‐quality sequences and mismatches, 4,694,613 effective tags (effective ratio: 93.32%) from the 38 samples were obtained. A total of 9494 OTUs were identified in all samples. After removing non‐fungal data, 1808 OTUs at 97% sequence identity were obtained. Of these, 724 OTUs were identified only at the fungal domain level. The average OTU for each samples was 238.58 (Table S2). The rarefaction curve tended to be flat, indicating that sequencing occurred in large enough quantities to represent the meta‐community (Figure S2).

**FIGURE 2:**
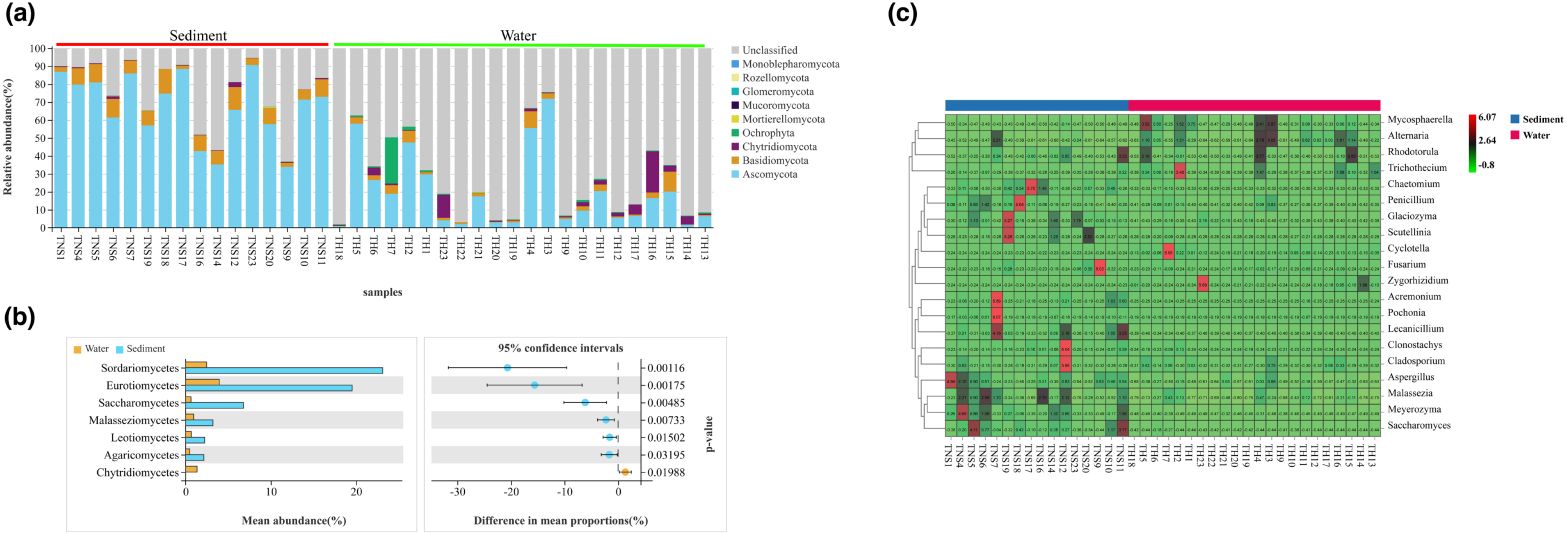
Fungal community composition of the water and sediments sampled from Hulun Lake. (a) Relative abundance of the main fungal phyla. (b) Response ratio at 95% confidence interval showing the classes that are enriched in the water and sediments. (c) Heat maps of species abundance at the genus level.

The taxonomically assigned OTUs from all samples belonged to nine phyla, 30 classes, 78 orders, 177 families, 307 genera, and 282 species. Four dominant phyla (relative abundance >1%) were identified, and these accounted for 26.96% and 75.49% of the phyla in water and sediment, respectively (Figure 2a). These dominant phyla had different relative abundances in water and sediment. Statistical tests confirmed significant differences between the water and sediment communities (Table S3). The class Chytridiomycetes (1.35% in water vs. 0.01% in sediment) was significantly abundant in water, whereas Sordariomycetes (2.47% in water vs. 23.12% in sediment), Eurotiomycetes (3.95% in water vs. 19.53% in sediment), Saccharomycetes (0.64% in water vs. 6.79% in sediment), Malasseziomycetes (0.94% in water vs. 3.21% in sediment), Leotiomycetes (0.67% in water vs. 2.24% in sediment), and Agaricomycetes (0.49% in water vs. 2.15% in sediment) were significantly abundant in sediments (Figure 2b). The relative abundance of Dothideomycetes was enriched between the water and sediments, but not significantly. At the genus level, four and eight genera exhibited relative abundances higher than 1% in water and sediment, respectively (Figure 2c).

**FIGURE 3:**
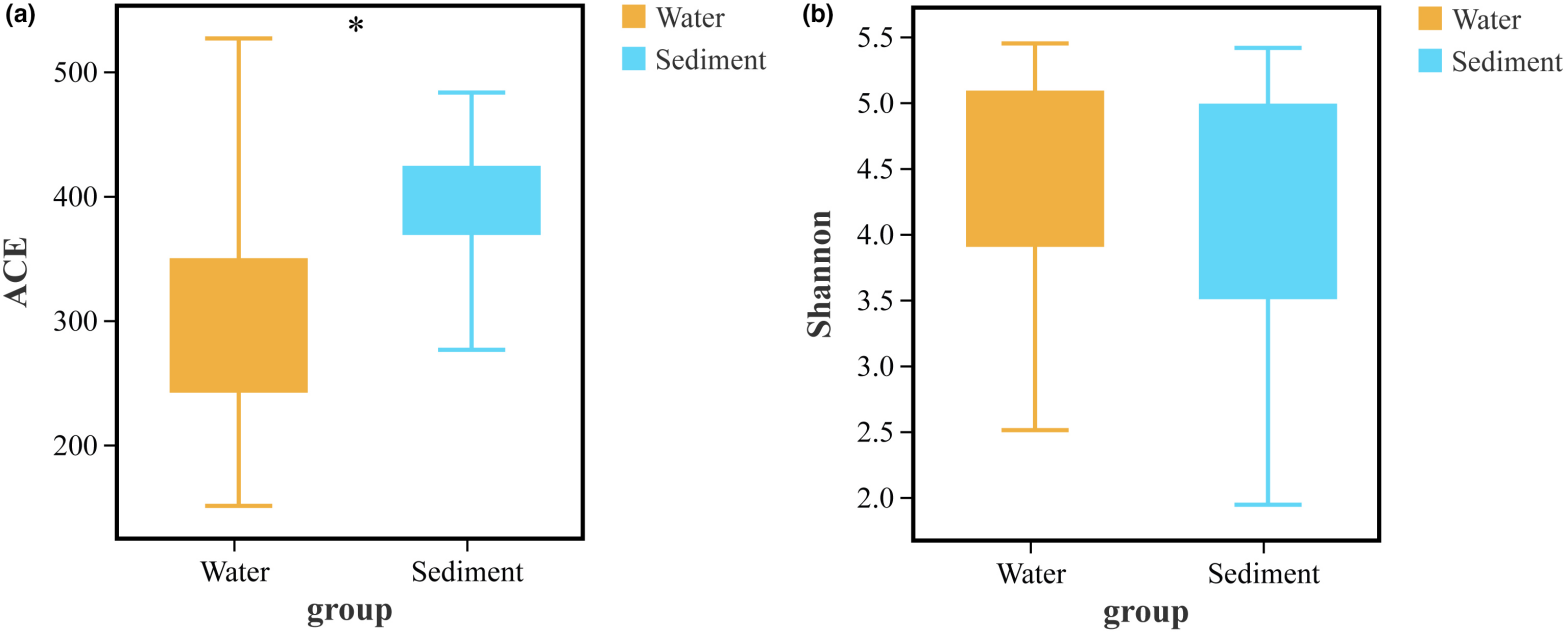
Wilcoxon text of α diversity indices of fungal communities: (a) Abundance‐based coverage estimator; (b) Shannon.

**FIGURE 4:**
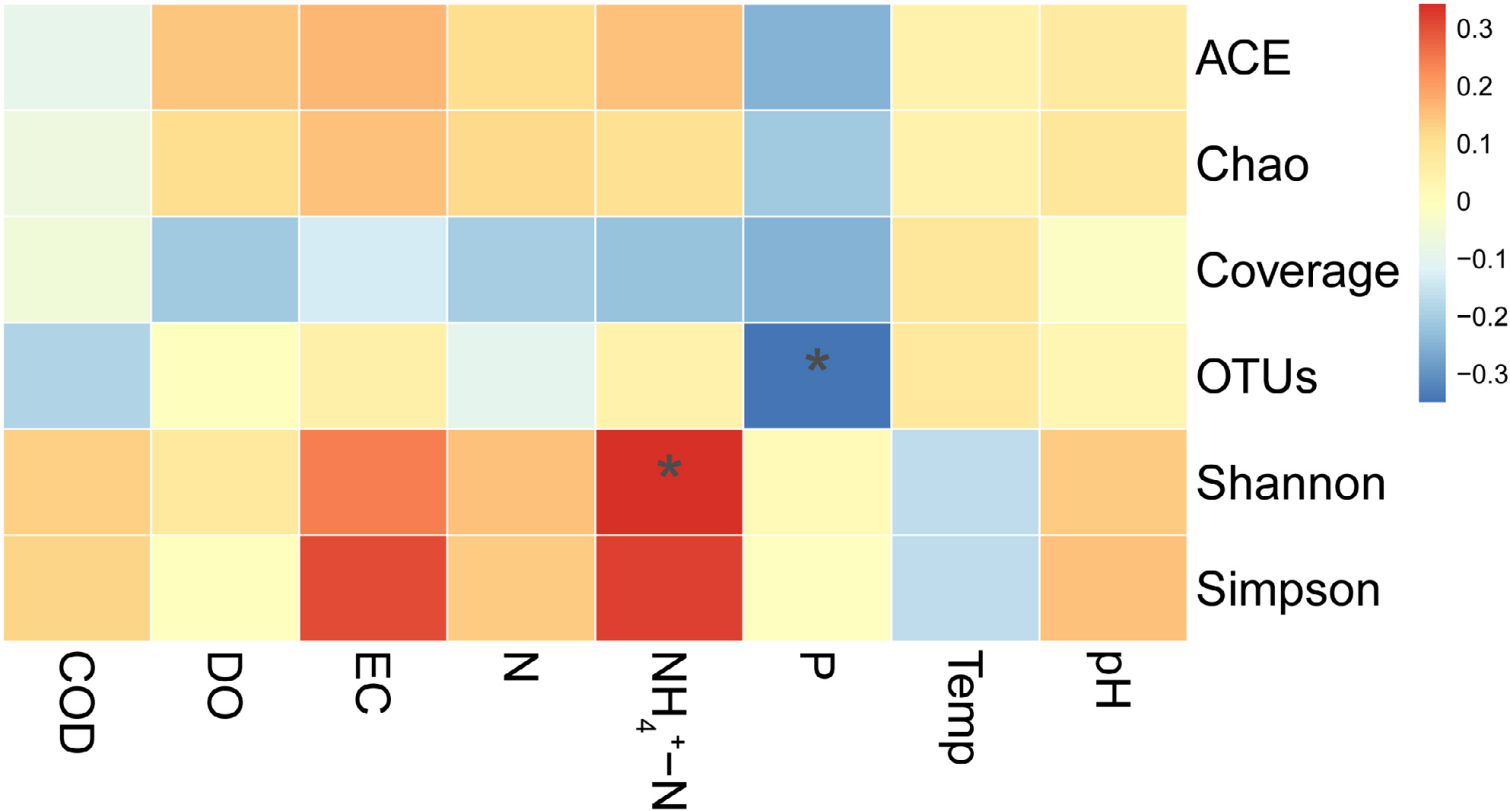
Correlation analysis of α diversity indices and environmental factors (*p < .05). COD, chemical oxygen demand; DO, dissolved oxygen; EC, electrical conductivity; N, total nitrogen; NH_4_^+^‐N, ammonia nitrogen; P, total phosphorus; Temp, temperature.

The distribution of the alpha diversity indices in Hulun Lake during water and sediment deposition was investigated (Figure 3). The abundance‐based coverage estimator index (Wilcoxon test, *p* < .05) exhibited significant differences between the water and sediment samples. However, the Shannon index (Wilcoxon test, *p* > .05) did not exhibit significant differences. The correlations between these factors are shown in Figure 4. NH_4_^+^‐N was significantly positively correlated with the Shannon index (*R* = .34, *p* < .05), while P was significantly negatively correlated with the OTUs (*R* = −.34, *p* < .05).

### 3.3 Overall patterns in fungal community structure and diversity

**FIGURE 5:**
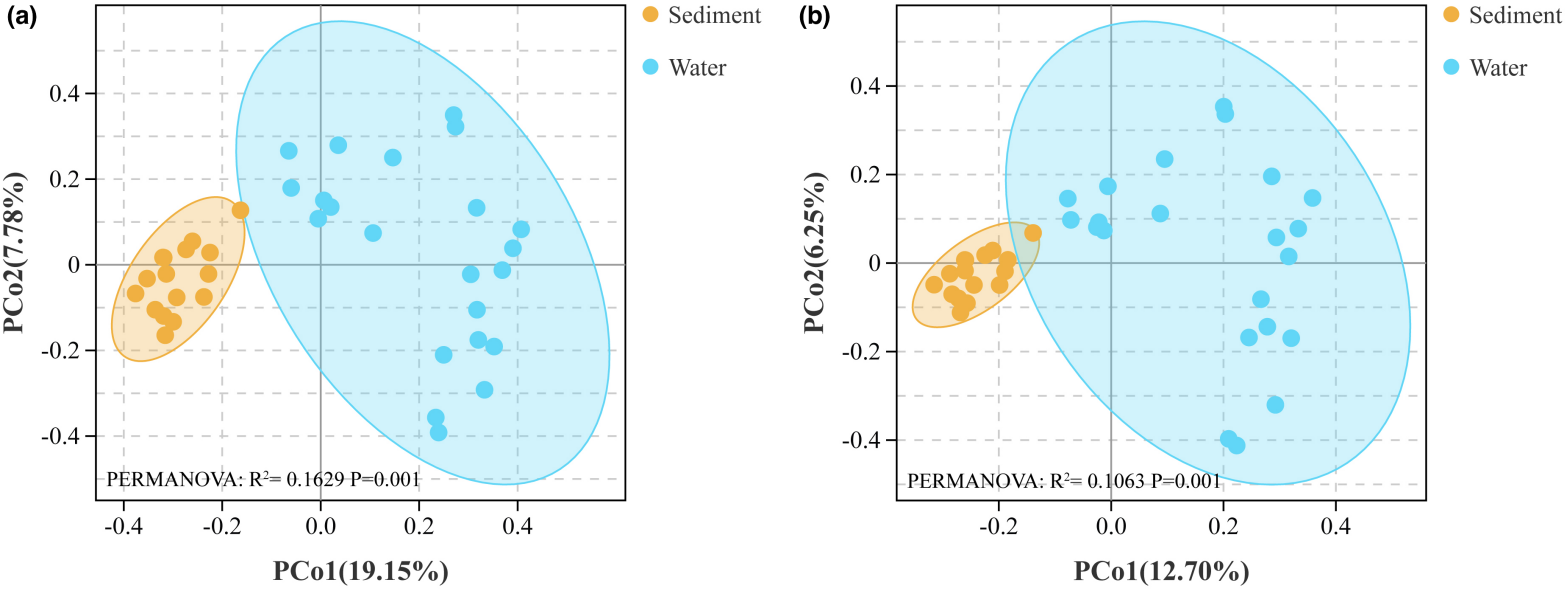
Principal co‐ordinate analysis of the fungal communities based on (a) Bray–Curtis distances and (b) Jaccard distances.

The PCoA based on the Bray–Curtis distance revealed a clear distinction in the fungal community structures between the water and sediment samples (Figure 5a). The PCoA based on Jaccard distance also showed a similar clustering pattern (Figure 5b). PERMANOVA and ANOSIM based on Bray–Curtis and Jaccard distances further confirmed significant differences between the water and sediment fungal communities (Table 1).

**TABLE 1:** Dissimilarity comparisons among water and sediment fungal communities using two non‐parametric statistical methods.

Distance matrix	Diffs	PERMANOVA		ANOSIM	
		*R* ^2^	*p*‐value	*R* ^2^	*p*‐value
Bray–Curtis distance	Water vs. sediment	.1629	.001	.7698	.001
Jaccard distance	Water vs. sediment	.1063	.001	.7698	.001

**FIGURE 6:**
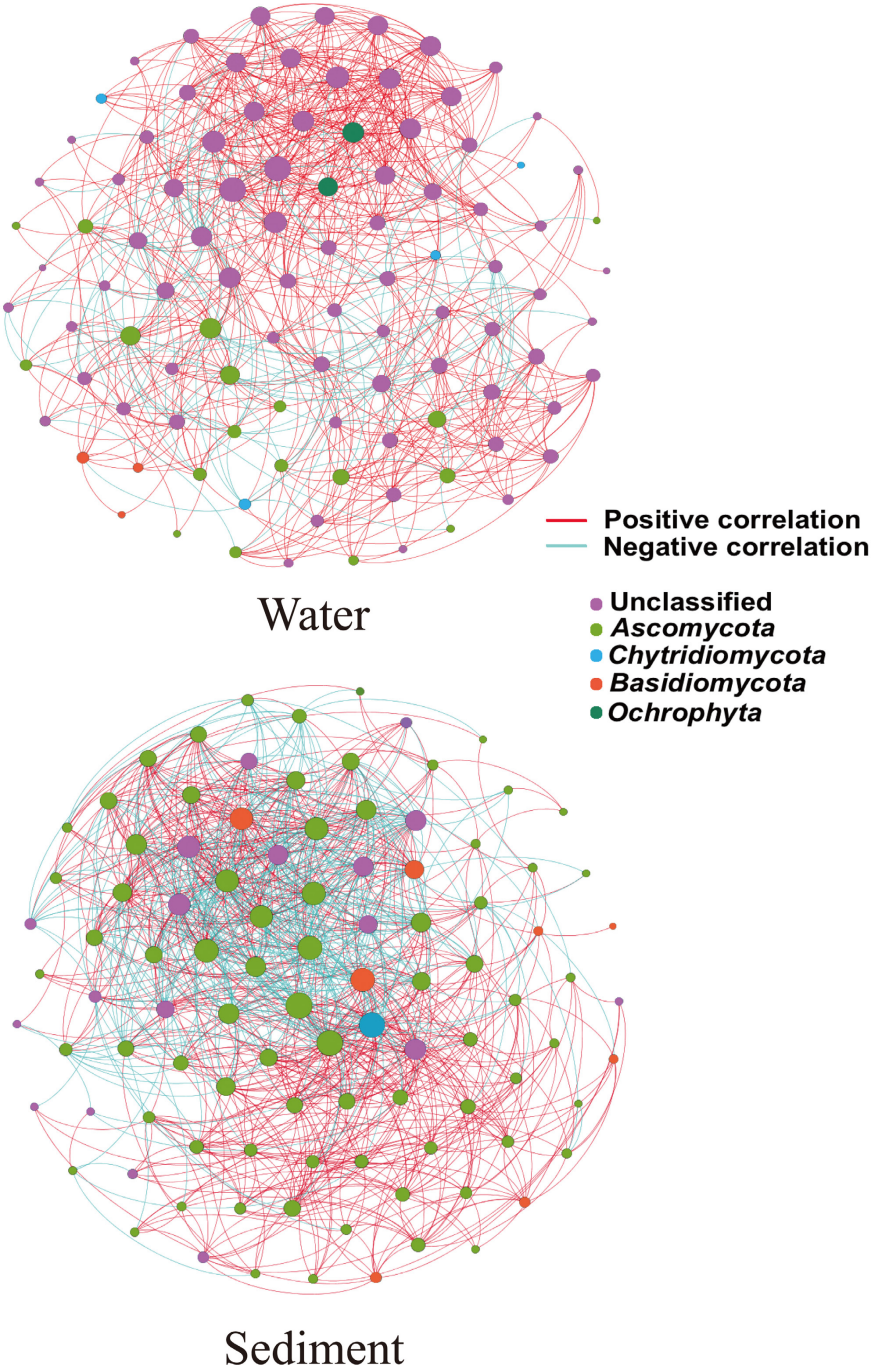
Network and correlation analysis of water‐ and sediment‐related fungal communities. Microbial correlation networks in water and sediments. Different phyla are represented by nodes in different colors. Node size represents the degree of operational taxonomic units.

Next, fungal networks were constructed between the water and sediment fungal communities. Most of the nodes in the network had many connections, whereas only a few nodes had a few connections (Table S4, Figure 6). Communities from the sediment showed a larger average degree, shorter average path length, and smaller modularity than those from water. However, water‐related communities showed a higher average clustering coefficient than sediment‐related communities. In conclusion, the microbial interactions in the sediments were more similar to those in water.

In the networks, the positive associations among species were predominant, with a ratio of positive to negative interactions of 5.84 in water and 2.11 in sediment (Table S4). The OTUs in the network mainly belonged to Ascomycota, Chytridiomycota, Basidiomycota and unclassified. Mantel tests were conducted to compare connectivity with the significance of the environmental factors in the entire network and major phyla in the water and sediments (Table S5). The whole network (*p* < .01) and unclassified (*p* < .05) in the water were significantly correlated with NH_4_^+^‐N. Ascomycota in water was significantly correlated with pH (*p* < .05). However, none of the environmental factors could be correlated with the variation in the sediment network structure.

### 3.4 Relationships between environmental factors and fungal community structure

**FIGURE 7:**
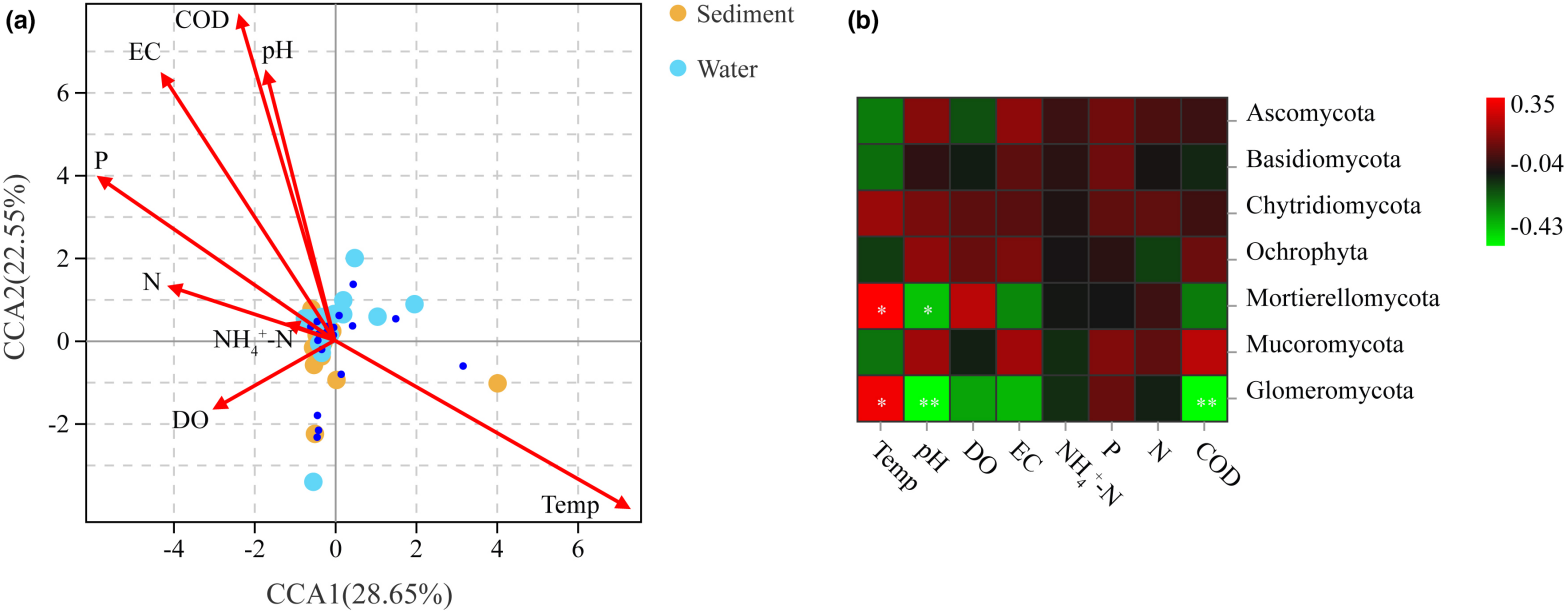
Environmental factor analysis. (a) Canonical correspondence analysis (CCA) based on the fungal communities operational taxonomic units (OTUs) level and environmental factors (red arrows). The top 20 most abundant classified fungal OTUs (97% sequence similarity) in the samples. The little blue represent the top 20 most abundant classified fungal OTUs. The direction of the arrow indicates the environmental factors associated with changes in the community structure, and the length of the arrow indicates the magnitude of the association. The percentage of variation explained by CCA1 and CCA2 is shown. (b) Pearson's correlation heat map analysis of environmental factors and species. Environmental factors are on the horizontal axis, species are on the vertical axis, and color indicates the strength of correlation.

To study the influence of environmental factors on the fungal community structure of Hulun Lake, we performed association analysis between fungi and environmental factors. After all the data were normalized, we carried out model screening for RDA and canonical correspondence analysis (CCA). As the axis length was 6.8144, we selected CCA for analysis. The ordination diagram showed that across all samples, the first two axes explained 51.2% of the variation in the fungal community structure (Figure 7a). Temperature, COD, EC, P, and pH showed strong effects on the fungal community structure (Figure 7b, Figure S3). Pearson's correlation analysis showed that some dominant fungal communities were significantly related to temperature, pH, and COD (Figure 7b). Mortierellomycota and Glomeromycota were positively correlated with temperature (*R* = .3535, *p* < .05 and *R* = .3535, *p* < .05, respectively), but negatively correlated with pH (*R* = −. 3335, *p* < .05 and *R* = −.4341, *p* < .01, respectively). Glomeromycota was also negatively correlated with COD (*R* = −.4347, *p* < .01).

### 3.5 Community assembly mechanisms in water and sediments

**FIGURE 8:**
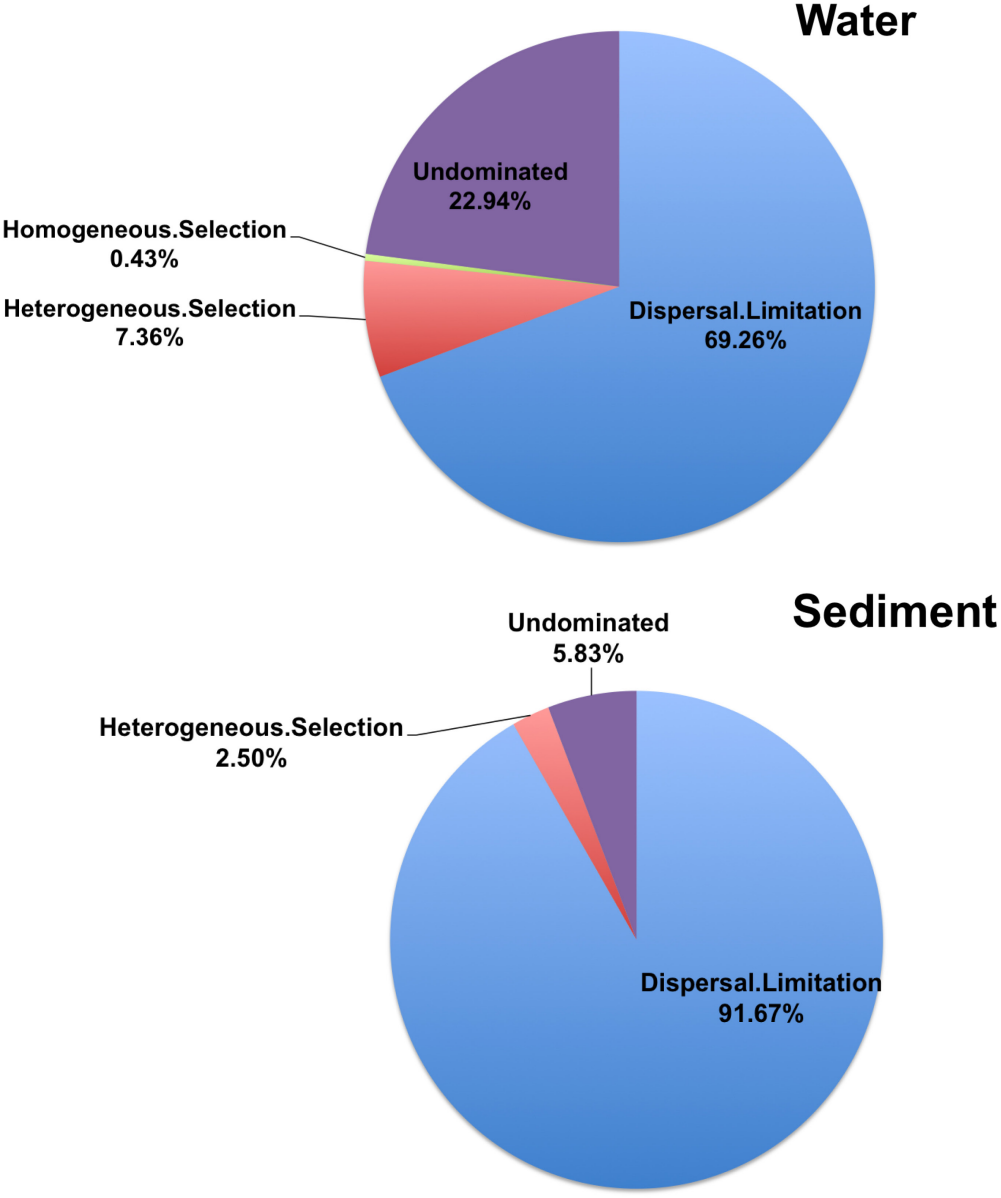
Community assembly mechanisms in water and sediments (Determinism: Homogeneous selection and Heterogeneous selection; Stochasticity: dispersal limitations and homogenizing dispersal; “undominated”).

To explore the relative contributions of the stochastic and deterministic processes to the composition of the water and sediment communities, we used a null model for the analysis. |βNTI| was < 2 in most water and sediment samples, suggesting that stochastic processes had a greater influence on this community (Figure S4). The results showed that dispersal limitation was dominant in water (69.26%), followed by undominated processes (22.94%). Furthermore, dispersal limitation was dominant in the sediments (91.67%) (Figure 8).

## 4 DISCUSSION

Understanding the main environmental factors affecting fungal communities in lake ecosystems could help in the generation and maintenance of fungal diversity in both water and sediments. The results of this study showed that the differences in the community structures between water and sediments were mainly caused by differences in fungal abundance. However, temperature, COD, EC, P, and pH were also found to have significant effects on fungal community structure. Stochastic processes were shown to play a dominant role in both water and sediment.

Lake ecological functions are based on lake microbial communities. Fungi, which are an important part of lake ecosystems, play a key role in degrading organic matter and causing disease (Alonso‐Monge et al., 2003; Eyles et al., 2006). In this study, there was no significant difference in fungal diversity between the water and sediment at the phylum level, but there was a significant difference in abundance, which may be related to the different habitats. Water and sediment represent different habitat types, each with unique characteristics (He et al., 2021). Sediment habitats are more complex than water bodies and some microhabitats are favorable for microbial growth as they provide shelter and nutrients (Boyd et al., 2007; Dockrey et al., 2014; Inskeep et al., 2004). Previous studies have suggested that habitat differentiation may lead to differences in the microbial communities in the water and sediments (Colman et al., 2016; Wang et al., 2014).

Numerous studies have demonstrated that fungal alpha diversity depends on different environmental variables (Liu et al., 2018; Sheng et al., 2019). For instance, salinity is the most important environmental factor affecting estuarine fungal community (Yang et al., 2021), whereas pH, Cd and Zn are closely related to fungi in eutrophic rivers (Yao et al., 2022). In this study, NH_4_^+^‐N and P were significantly correlated with the Shannon index and OTUs, respectively. These results were different from those of previous studies on the bacterial communities in Hulun Lake (Shang et al., 2022). This suggests a major difference between bacteria and fungi. Both Shannon index and OTUs are indicators of species diversity (Chao & Shen, 2003). We conjectured that the fungi related to N transformation in the lake were more diverse and increased with the increase of ammonia nitrogen, while the fungi related to P transformation were rare, which also indicated that the P transformation ability of Hulun Lake was to a certain extent weak. In addition, some abiotic factors may play important roles in driving fungal alpha diversity.

Eight phyla were identified in the samples in this study; among these, Ascomycota, Basidiomycota, and Chytridiomycota were the main phyla. This is inconsistent with previous findings, wherein the abundance of Basidiomycota was found to be low in aquatic ecosystems (Bärlocher & Boddy, 2016; Grossart & Rojas‐Jimenez, 2016). The dominance of Basidiomycota in our study may be related to the inclusion of sediment samples and primer bias. Previous studies have found that primer bias can lead to high abundance of some taxa during amplification (Ihrmark et al., 2012). Studies have shown that most Basidiomycota are saprophytes, which have a relatively stable response to environmental stress (Jia et al., 2020; Ventorino et al., 2016). As Basidiomycota can resist environmental stress, they may play an important role in maintaining the stability of lake ecosystems. Metcalf et al. (2016) reported that Ascomycota as a post‐putrefaction phylum (Metcalf et al., 2016). In this study, the abundance of Ascomycota in the sediment was much higher than that in water. This coincided with a more complex sediment environment. The upper sediment surface (~5 mm) is usually oxic (high water content, >95%) and has high organic matter content (Wurzbacher et al., 2016). Thus, the complex sediment environment serves as a fungal spore bank. Kagami et al. (2014) found that Chytridiomycota play an important role in water bodies as they mainly feed on dead aquatic plants, but are grazed upon by zooplankton (Kagami et al., 2014). Fungi that live in different habitats vary as they adapt to the different conditions, but all play important ecological roles (Bärlocher & Boddy, 2016; Chen et al., 2022; Raja et al., 2008). Meanwhile, many unclassified fungi were also found in our study. There may be two main reasons for this. First, the ITS2 sequences of some fungal groups in the public database are lacking, which results in the alignment of these groups as non‐fungi (Khomich et al., 2018). Second, the current fungal classification database itself is not complete. This implies that there is need to pay more attention to studies related to fungi in the future.

The fungus‐specific primers used in this study were not tested for all fungal members. Meanwhile, we found that Ochrophyta and unclassified represented a large proportion, further indicating that the primer combination ITS3_KYO2/ITS4 was not fungus‐specific in aquatic environment. This substantially reduced the effective sequencing depth for fungal taxa. Cryptomycota is known to exist in freshwater environments, as well as in marine and soil ecosystems (Khomich et al., 2017). However, in our samples, we did not detect the presence of these fungi, and we suspect that this may be due to primer bias.

Microbial networks provide important insights into microbial communities (He et al., 2021). Understanding the interactions between microbial communities can reveal the dynamics of complex microbial community structures (Barberan et al., 2012). Our results show that both water and sediments have very high connectivity, are dominated by positive interactions, and have similar interaction patterns. These findings differ from those of previous research on hot springs (He et al., 2021). This may possibly be related to the different environments in the two communities. The temperature in hot springs is higher all the year‐round, whereas the temperature difference in Hulun Lake is larger, which may affect the interactions between communities to some extent. Banerjee et al. (2018) found that keystone species influence the structure and function of the microbial communities (Banerjee et al., 2018). In our study, Ascomycota dominated the network. The positive interaction between the Ascomycota suggests that they may have a synergistic effect (Barberan et al., 2012). In addition, Ascomycota were more abundant in the sediments than in water, indicating that they were favored by a saprophytic environment (Metcalf et al., 2016). Chytridiomycota and Basidiomycota, the major members of the fungal community, also dominated the network, but their abundance was lower than that of the ascomycetes, which is consistent with previous studies (Khomich et al., 2017; Shearer et al., 2006).

Microbial communities are closely related to their environments (Abirhire et al., 2015; Zeng, Jia, et al., 2019). Previous studies have shown that environmental factors such as pH, EC, N, and P can influence fungal community structures (Cudowski et al., 2015; Reich et al., 2017). In our study, temperature, COD, EC, P, and pH exhibited strong effects on the fungal community structure. This observation is slightly different from the important environmental factors affecting bacterial community distribution in Hulun Lake identified in our previous study (Shang et al., 2022); only temperature and pH are common environmental factors affecting both bacterial and fungal community distribution. Although we identified some environmental factors that influence fungal communities, we are still unable to explain all the observed changes. These unexplained variations may be due to undetected environmental factors. Therefore, in future studies, it will be necessary to analyze other environmental factors.

The deterministic process based on niche theory suggests that microbial diversity is mainly controlled by environmental filtration and interactions between organisms, whereas the stochastic process based on neutral theory emphasizes the role of diffusion and ecological drift (Zhang, Yin, et al., 2021). Previous studies have shown that both stochastic and deterministic processes affect the development of microbial communities (Ortiz‐Alvarez et al., 2020; Sun et al., 2021). To better understand the mechanisms of microbial ecology, the relative effects of the random and deterministic assembly processes must be quantified (Feng et al., 2018). In this study, stochastic processes were dominant in both water and sediment. The dispersal limitation was dominant in water (69.26%), and in sediment, its proportion was as high as 91.67%. It has been previously reported that bacterial communities in freshwater ecosystems are mainly formed by deterministic biological and abiotic processes (Zhang, Yin, et al., 2021). However, because fungi are more complex in structure and function, which can promote niche adaptation and reduce environmental filtering effects at the community level, stochastic processes dominate (Massana & Logares, 2013). Similar patterns have been observed in lakes in Antarctica and Tibet (Liu, Liu, et al., 2020; Logares et al., 2018). Lakes, including Hulun Lake, are highly fluidized ecosystems (Meyerhof et al., 2016). A high flow rate can overwhelm the selection process (Konopka et al., 2015). In addition, owing to the higher abundance of fungi in the sediments and their ability to provide more nutrients, there was a stronger ecological drift (Zeng, Lin, et al., 2019). Some studies have found that random assembly processes play a dominant role under high microbial diversity, whereas deterministic processes play a dominant role under low microbial diversity, which may be related to the reduction in functional microorganisms in the microbial community (Xun et al., 2019). Another reason for the higher dispersal limitation in sediments than in water may be that sediments have lower diffusion capacity and lower regional connectivity than water, which reduces the likelihood of active diffusion and enhances the stochastic effect (Liu, Zhu, et al., 2020; Zinger et al., 2014).

## 5 CONCLUSION

In this study, fungal community structures and abiotic factors were investigated in Lake Hulun, which is in northern China and seasonally frozen. The main fungal communities identified comprised of Ascomycota, Basidiomycota and Chytridiomycota as well as many unclassified fungi. High‐throughput sequencing analysis showed that there was no significant difference in the fungal diversity between water and sediment, although there was a significant difference in fungal abundance. The results showed that the temperature, COD, EC, P, and pH strongly affected fungal community structures. In addition, stochastic processes were dominant in both water and sediment. In the future, continuous dynamic tracking of fungal microbial communities across seasons should be performed to further increase our understanding of the fungal community structures and functions in near‐extreme environments.

## AUTHOR CONTRIBUTIONS

**Yongquan Shang:** Data curation (equal); formal analysis (equal); project administration (equal); writing – original draft (equal); writing – review and editing (equal). **Xiaoyang Wu:** Formal analysis (equal); project administration (equal); writing – review and editing (equal). **Xibao Wang:** Project administration (equal). **Huashan Dou:** Project administration (supporting). **Qinguo Wei:** Project administration (supporting). **Shengchao Ma:** Formal analysis (supporting). **Guolei Sun:** Formal analysis (supporting). **Lidong Wang:** Formal analysis (supporting). **Weilai Sha:** Conceptualization (supporting). **Honghai Zhang:** Funding acquisition (lead); project administration (equal).

## FUNDING INFORMATION

This work was supported by the National Natural Science Foundation of China (31872242, 32070405, 32001228, and 32170530).

## CONFLICT OF INTEREST

The authors declare that the research was conducted in the absence of any commercial or financial relationships that could be construed as a potential conflict of interest.

## Supporting information


Figure S1

Figure S2

Figure S3

Figure S4

Appendix S1

Data S1

## Data Availability

The raw sequencing data have been deposited in the NCBI SRA database under the BioProject accession number PRJNA781770 (https://dataview.ncbi.nlm.nih.gov/object/PRJNA781770?reviewer=8910vtfimi25fjk1cum5ege9op).
