# Supplementary figures and images for: Environmental factors and stochasticity affect the fungal community structures in the water and sediments of Hulun Lake, China
Source: Ecol Evol. 2022 Nov 18;12(11):e9510. doi: 10.1002/ece3.9510 (PMC9674472; doi:10.1002/ece3.9510)

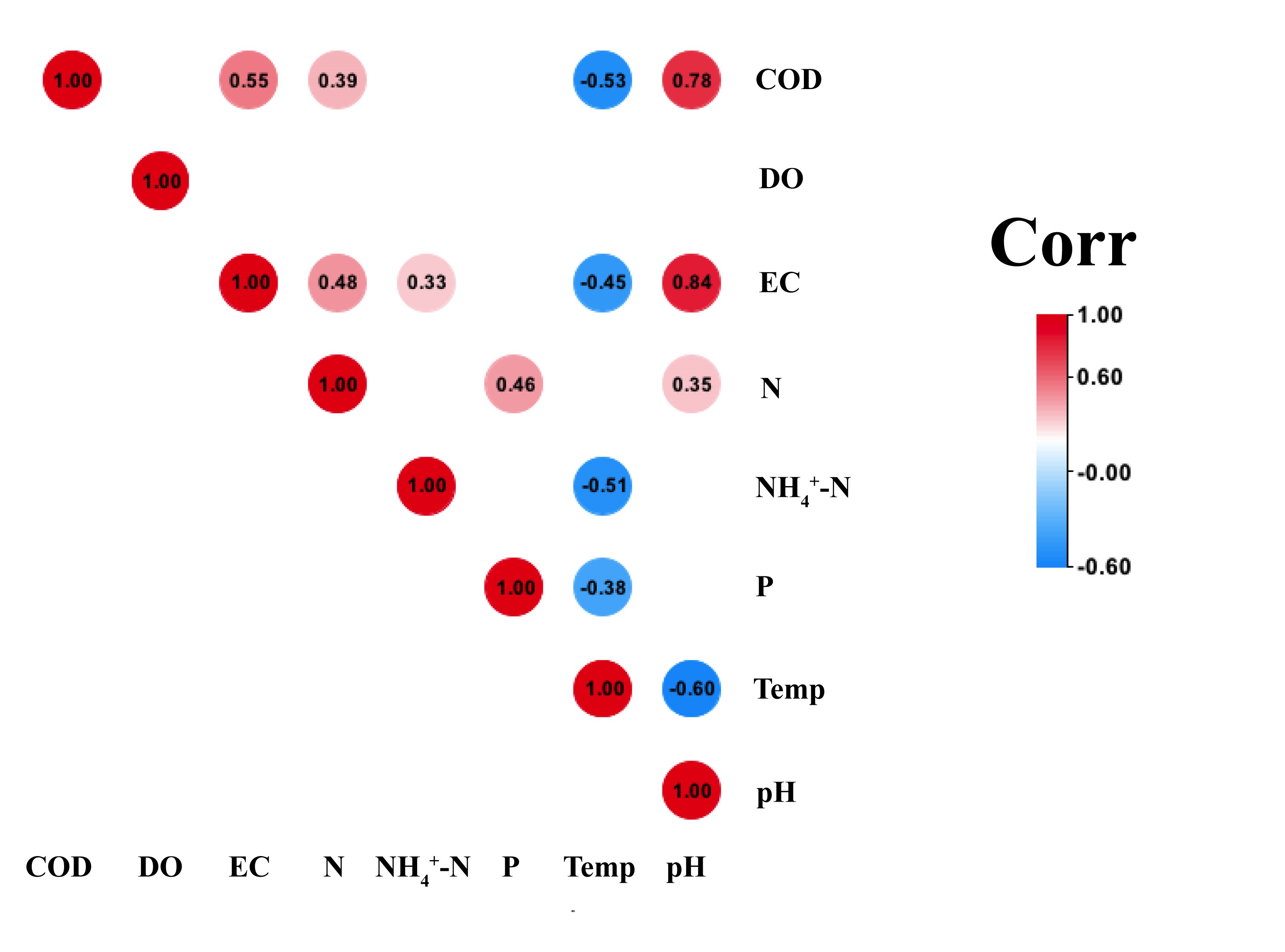

Supplement: Supplementary file 1 — Figure S1 [file ECE3-12-e9510-s002.jpg]

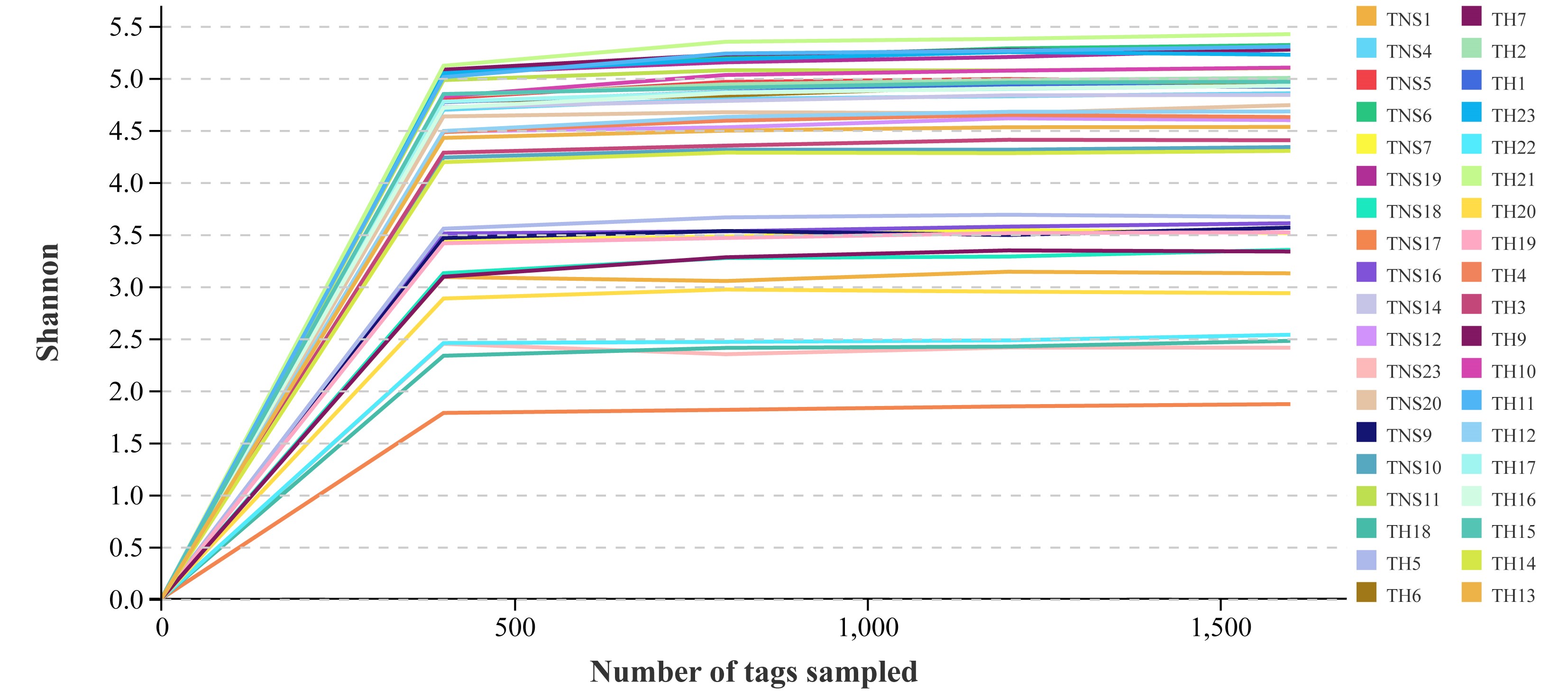

Supplement: Supplementary file 2 — Figure S2 [file ECE3-12-e9510-s006.jpg]

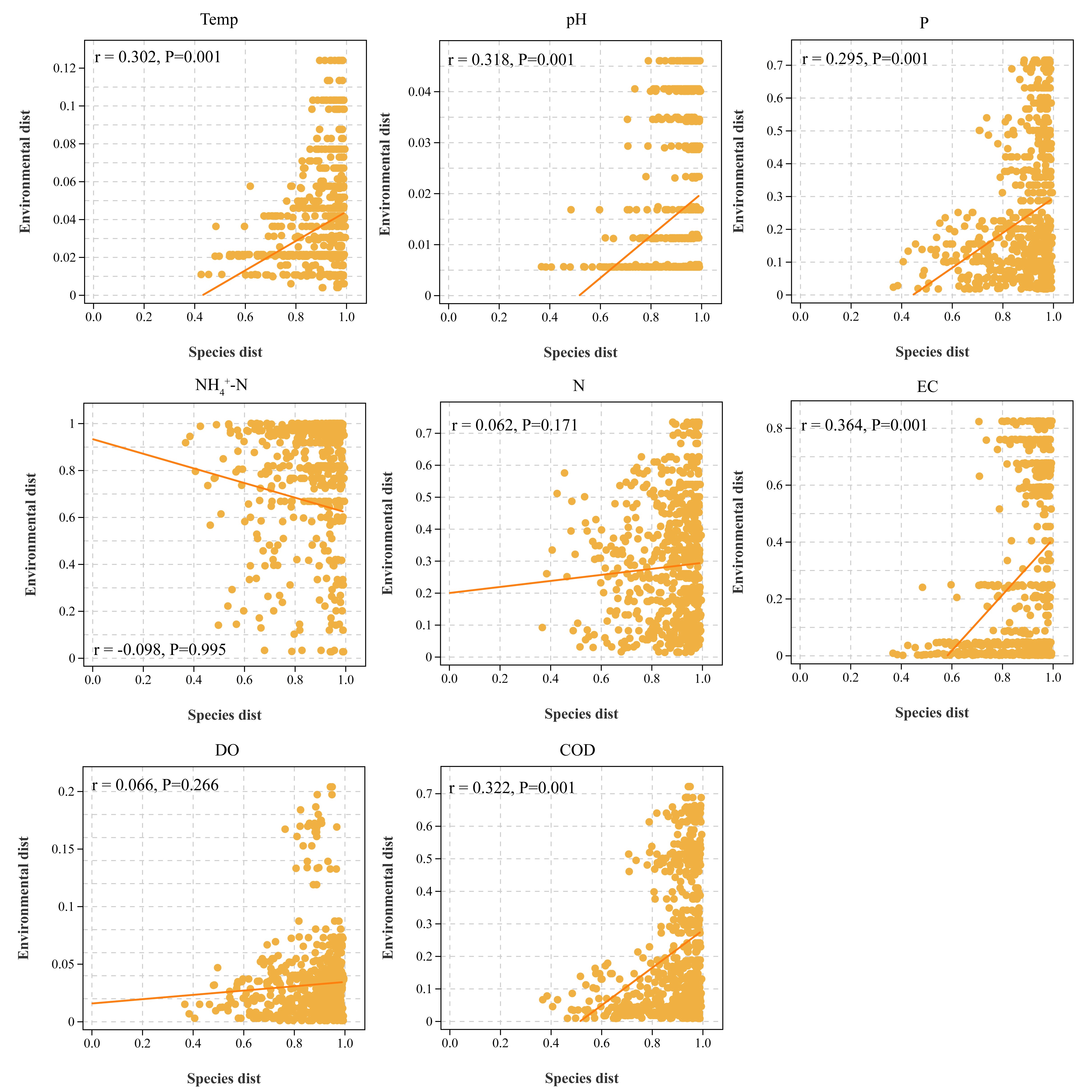

Supplement: Supplementary file 3 — Figure S3 [file ECE3-12-e9510-s001.jpg]

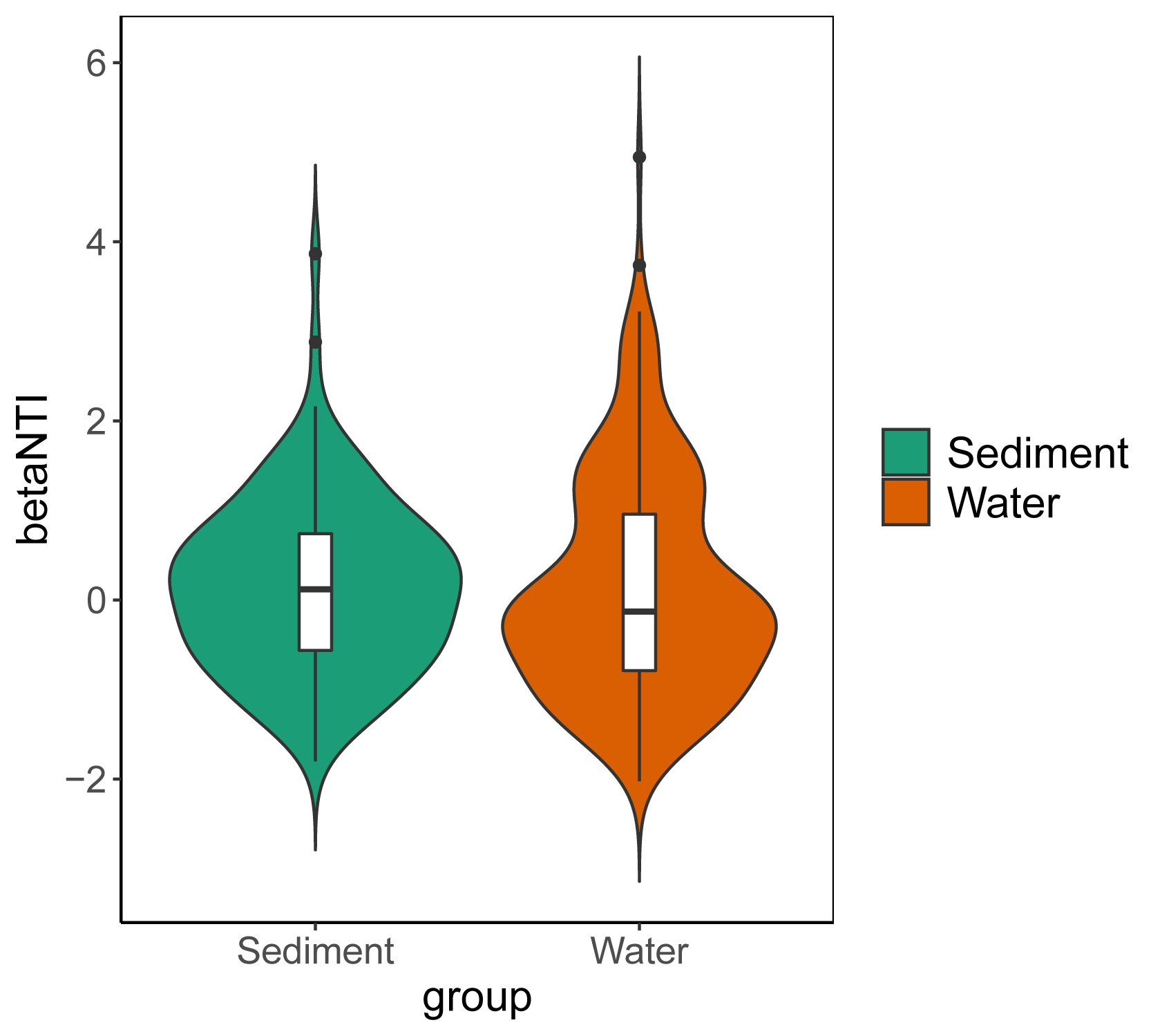

Supplement: Supplementary file 4 — Figure S4 [file ECE3-12-e9510-s003.jpg]
