# Supplementary material for: Environmental factors and stochasticity affect the fungal community structures in the water and sediments of Hulun Lake, China
Source: Ecol Evol. 2022 Nov 18;12(11):e9510. doi: 10.1002/ece3.9510 (PMC9674472; doi:10.1002/ece3.9510)
Supplement: Supplementary file 5 — Appendix S1 [file ECE3-12-e9510-s005.doc]

***Supplementary Material***

The following supplementary information appendix covers two main topics. The first section describes high-throughput sequencing. The second section is supplementary tables and figures.

High-throughput sequencing

To get high-quality clean reads, raw reads were further filtered using FASTP (Chen et al., 2018) (version 0.18.0). Paired end clean reads were merged as raw tags using FLASH (version 1.2.11) with a minimum overlap of 10bp and mismatch error rates of 2% (Magoč et al., 2011). Noisy sequences of raw tags were filtered by QIIME (version 1.9.1 ) to obtain the high-quality clean tags (Bokulich et al., 2013). Reference-based chimera checking using UCHIME algorithm were performed to remove the chimeric tags (Edgar et al., 2011). The effective tags were clustered into operational taxonomic units (OTUs) of ≥ 97 % similarity using UPARSE (version 9.2.64) pipeline (Edgar et al., 2013). The taxonomic identities of the gene sequences for each ITS were assigned by BLAST against the UNITE fungal ITS database (version 7). The tag sequences with the highest abundance were selected as representative sequences within each cluster. Rarefaction curves were created using Origin software.

**References:**

Bokulich, Nicholas A., et al. Quality-filtering vastly improves diversity estimates from Illumina amplicon sequencing. Nature methods 10.1 (2013): 57-59.

Chen S, Zhou Y, Chen Y, et al. fastp: an ultra-fast all-in-one FASTQ preprocessor[J]. bioRxiv, 2018: 274100.

Edgar, Robert C. UPARSE: highly accurate OTU sequences from microbial amplicon reads. Nature methods 10.10 (2013): 996-998.

Edgar, Robert C., et al. UCHIME improves sensitivity and speed of chimera detection. Bioinformatics 27.16 (2011): 2194-2200.

Magoč T, Salzberg S L. FLASH: fast length adjustment of short reads to improve genome assemblies. Bioinformatics 27.21 (2011): 2957-2963.

| Sample code | Description of samples | | | Environmental parameters | | | | | | | |
| --- | --- | --- | --- | --- | --- | --- | --- | --- | --- | --- | --- |
|  | Name | GPS | Sample Type | Temperature(℃) | pH | DO (mg/L) | EC(us/cm) | NH_4_^+^-H(mg/L) | P(mg/L) | N(mg/L) | COD(mg/L) |
| 1 | TNS1\TH1 | N49.3095°E117.68963° | Sediment & Water | 23.0 | 9.0 | 7.60 | 1584 | 0.20 | 0.27 | 1.8 | 62 |
| 2 | TH2 | N49.2519°E117.61475° | Water | 23.0 | 9.0 | 7.38 | 1589 | 0.72 | 0.28 | 3.0 | 61 |
| 3 | TH3 | N49.22355°E117.49542° | Water | 23.0 | 9.0 | 7.34 | 1582 | 0.02 | 0.23 | 1.2 | 50 |
| 4 | TNS4\TH4 | N49.1615°E117.41966° | Sediment & Water | 23.0 | 8.9 | 7.56 | 1606 | 0.46 | 0.22 | 1.0 | 57 |
| 5 | TNS5\TH5 | N48.92639°E117.13842° | Sediment & Water | 23.5 | 9.0 | 7.56 | 1697 | 3.00 | 0.30 | 3.7 | 57 |
| 6 | TNS6\TH6 | N48.85732°E117.01166° | Sediment & Water | 24.0 | 9.0 | 7.87 | 1721 | 11.30 | 0.22 | 1.6 | 44 |
| 7 | TNS7\TH7 | N48.77977°E°117.05325 | Sediment & Water | 24.0 | 9.1 | 8.19 | 1731 | 0.02 | 0.20 | 1.2 | 59 |
| 9 | TNS9\TH9 | N°48.71831E°117.30044 | Sediment & Water | 24.0 | 9.1 | 7.61 | 1736 | 0.02 | 0.19 | 2.0 | 70 |
| 10 | TNS10\TH10 | N°48.95555E°117.47722 | Sediment & Water | 24.0 | 9.1 | 7.74 | 1732 | 45.00 | 0.19 | 2.0 | 63 |
| 11 | TNS11\TH11 | N°48.95611E°117.73694 | Sediment & Water | 24.0 | 9.0 | 7.64 | 1741 | 1.31 | 0.18 | 3.4 | 54 |
| 12 | TNS12\TH12 | N°48.96851E°117.63537 | Sediment & Water | 24.0 | 9.1 | 7.60 | 1740 | 0.15 | 0.22 | 1.7 | 59 |
| 13 | TH13 | N°48.20388E°117.86111 | Water | 24.0 | 9.1 | 7.68 | 1707 | 0.76 | 0.24 | 2.6 | 64 |
| 14 | TNS14\TH14 | N°49.14039E°117.7512 | Sediment & Water | 25.8 | 8.8 | 8.18 | 1048 | 0.10 | 0.27 | 0.9 | 54 |
| 15 | TH15 | N°48.85655E°117.22237 | Water | 24.0 | 9.0 | 7.68 | 1710 | 0.93 | 0.29 | 1.7 | 58 |
| 16 | TNS16\TH16 | N°49.00232E°117.44392 | Sediment & Water | 25.0 | 9.0 | 8.43 | 1718 | 1.23 | 0.30 | 3.3 | 59 |
| 17 | TNS17\TH17 | N°49.07006E°117.5942 | Sediment & Water | 25.0 | 9.0 | 8.09 | 1738 | 0.02 | 0.29 | 3.9 | 55 |
| 18 | TNS18\TH18 | N°49.30447E°118.04613 | Sediment & Water | 25.5 | 8.4 | 8.20 | 168 | 0.19 | 0.10 | 1.4 | 19 |
| 19 | TNS19\TH19 | N°49.42896E°117.78493 | Sediment & Water | 26.3 | 8.3 | 7.52 | 238 | 0.02 | 0.09 | 1.3 | 20 |
| 20 | TNS20\TH20 | N°48.75912E°117.04425 | Sediment & Water | 28.0 | 8.3 | 7.08 | 336 | 0.02 | 0.26 | 2.0 | 13 |
| 21 | TH21 | N°48.34636E°117.48103 | Water | 26.5 | 8.9 | 10.70 | 1461 | 0.02 | 0.18 | 3.8 | 80 |
| 22 | TH22 | N°47.96489E°117.71295 | Water | 26.0 | 8.5 | 7.44 | 354 | 0.02 | 0.05 | 0.6 | 44 |
| 23 | TNS23\TH23 | N°48.96077E°117.74629 | Sediment & Water | 29.5 | 8.8 | 7.18 | 446 | 0.02 | 0.05 | 0.6 | 31 |

**Tables**

**Table S1**. Description of samples point and environmental parameters of Hulun lake investigated in this study.

Note: Temp, temperature; DO, dissolved oxygen; EC, conductivity; NH4+-N, ammonia nitrogen; P, total phosphorus; N, total nitrogen; COD, chemical oxygen demand.

**Table S2. Statistical table of OTUs and Tags of different samples.**

| Sample Name | Raw PE | Clean Tags | Effective Ratio (%) | Fungi_Tag number | Fungi_ OTU number |
| --- | --- | --- | --- | --- | --- |
| TH1 | 200024 | 188896 | 94.437 | 6012 | 190 |
| TH2 | 183577 | 173882 | 94.719 | 15718 | 251 |
| TH3 | 187015 | 177462 | 94.892 | 7762 | 208 |
| TH4 | 186820 | 176157 | 94.292 | 7651 | 179 |
| TH5 | 191463 | 181357 | 94.722 | 8041 | 176 |
| TH6 | 194333 | 182950 | 94.143 | 7488 | 299 |
| TH7 | 189060 | 176361 | 93.283 | 8420 | 346 |
| TH9 | 195754 | 183208 | 93.591 | 21287 | 328 |
| TH10 | 196681 | 181965 | 92.518 | 18702 | 400 |
| TH11 | 194907 | 177901 | 91.275 | 16011 | 400 |
| TH12 | 191358 | 171521 | 89.634 | 13922 | 203 |
| TH13 | 192055 | 177887 | 92.623 | 20982 | 209 |
| TH14 | 168342 | 152184 | 90.402 | 31824 | 227 |
| TH15 | 190131 | 174002 | 91.517 | 5312 | 162 |
| TH16 | 146424 | 136970 | 93.543 | 3638 | 175 |
| TH17 | 101002 | 94130 | 93.196 | 2419 | 131 |
| TH18 | 92268 | 86830 | 94.106 | 20751 | 219 |
| TH19 | 99863 | 93339 | 93.467 | 13651 | 294 |
| TH20 | 97802 | 90844 | 92.886 | 8855 | 206 |
| TH21 | 97169 | 92929 | 95.636 | 1946 | 201 |
| TH22 | 101550 | 95970 | 94.505 | 26207 | 265 |
| TH23 | 46493 | 43273 | 93.074 | 4813 | 135 |
| TNS1 | 101120 | 93531 | 92.495 | 56095 | 212 |
| TNS4 | 97326 | 92825 | 95.375 | 9994 | 240 |
| TNS5 | 100132 | 94116 | 93.992 | 17513 | 258 |
| TNS6 | 99376 | 94580 | 95.174 | 5309 | 253 |
| TNS7 | 96895 | 90943 | 93.857 | 60634 | 268 |
| TNS9 | 99640 | 93629 | 93.967 | 15860 | 81 |
| TNS10 | 102107 | 90210 | 88.348 | 20820 | 235 |
| TNS11 | 100516 | 94973 | 94.485 | 23838 | 234 |
| TNS12 | 100700 | 93091 | 92.444 | 48604 | 252 |
| TNS14 | 96293 | 91276 | 94.79 | 7195 | 194 |
| TNS16 | 98775 | 93129 | 94.284 | 10913 | 241 |
| TNS17 | 100418 | 95446 | 95.049 | 38462 | 252 |
| TNS18 | 101532 | 94977 | 93.544 | 73904 | 301 |
| TNS19 | 97372 | 91855 | 94.334 | 15390 | 243 |
| TNS20 | 94676 | 86539 | 91.405 | 56288 | 324 |
| TNS23 | 99524 | 93475 | 93.922 | 33197 | 274 |
| Average | 132381.39 | 123542.45 | 93.42 | 20142.84 | 238.58 |

**Table S3**. Welch’s t tests based on different distances of microbial communities for water and sediments.

| Water-sediment | P-value |
| --- | --- |
| Bray | 0.0064 |
| Jaccard | 0.0013 |

**Table S4**. Topological properties of the microbial phylogenetic molecular networks of water and sediment.

| Network indexes | water | sediment |
| --- | --- | --- |
| Similarity threshold | 0.50 | 0.50 |
| Network size^a^ | 101.00 | 100.00 |
| Total links^b^ | 792.00 | 1039.00 |
| Average Degree^c^ | 15.68 | 20.78 |
| Average Path Length^d^ | 2.28 | 1.96 |
| Average clustering coefficient^e^ | 0.26 | 0.26 |
| Modularity^f^ | 0.44 | 0.27 |
| Positive interaction / Negative interaction | 5.84 | 2.11 |

a Number of nodes

b Number of edges

c Average degree refers to the average connectivity for nodes in the whole network. Higher average degree means a more complex network.

d Average path length is similar to average geodesic distance. A smaller average path length all the nodes in the network are closer.

e Average clustering coefficient is the average clustering coefficient of nodes in the whole network. Higher Average clustering coefficient means more tightened network structure.

f Modularity measures how well a network is able to be separated into modules.

**Table S5**. Mantel tests were used to explore the effect of environmental factors on network structure and major phyla in water and sediments.

|  |  | Temp | pH | DO | EC | NH_4_^+^-N | P | N | COD |
| --- | --- | --- | --- | --- | --- | --- | --- | --- | --- |
| Water | Whole network | 0.782 | 0.773 | 0.878 | 0.892 | **0.006 **** | 0.81 | 0.852 | 0.926 |
|  | Ascomycota | 0.652 | **0.03 *** | 0.56 | 0.232 | 0.431 | 0.778 | 0.892 | 0.022 |
|  | Chytridiomycota | 0.916 | 0.515 | 0.748 | 0.513 | 0.1 | 0.677 | 0.988 | 0.518 |
|  | Basidiomycota | 0.607 | 0.264 | 0.876 | 0.297 | 0.138 | 0.569 | 0.918 | 0.549 |
|  | Ochrophyta | 0.776 | 0.494 | 0.658 | 0.427 | 0.415 | 0.178 | 0.131 | 0.924 |
|  | Unclassified | 0.695 | 0.34 | 0.902 | 0.405 | **0.022 *** | 0.51 | 0.751 | 0.369 |
| Sediment | Whole network | 0.807 | 0.171 | 0.81 | 0.294 | 0.304 | 0.267 | 0.861 | 0.318 |
|  | Ascomycota | 0.543 | 0.226 | 0.653 | 0.312 | 0.135 | 0.152 | 0.695 | 0.322 |
|  | Chytridiomycota | 0.381 | 0.269 | 0.513 | 0.5 | 0.506 | 0.867 | 0.948 | 0.203 |
|  | Basidiomycota | 0.194 | 0.678 | 0.461 | 0.503 | 0.195 | 0.147 | 0.158 | 0.499 |
|  | Unclassified | 0.336 | 0.2 | 0.056 | 0.109 | 0.976 | 0.137 | 0.139 | 0.277 |

Note： ***** P＜0.05；****** P＜0.01
